# Planar carbon electrodes for real-time quantification of hydrogen sulfide release from cells†

**DOI:** 10.1039/d2sd00179a

**Published:** 2022-12-01

**Authors:** Jackson R. Hall, James B. Taylor, Taron M. Bradshaw, Mark H. Schoenfisch

**Affiliations:** a Department of Chemistry, The University of North Carolina at Chapel Hill Chapel Hill North Carolina 27599 USA schoenfisch@unc.edu; b Division of Pharmacoengineering and Molecular Pharmaceutics, UNC Eshelman School of Pharmacy Chapel Hill NC 27599 USA

## Abstract

A planar electrode system was developed to permit the real-time, selective detection of hydrogen sulfide (H_2_S) from stimulated cells. Planar carbon electrodes were produced *via* stencil printing carbon ink through a laser cut vinyl mask. Electrodes were preconditioned using a constant potential amperometry methodology to prevent sensor drift resulting from elemental sulfur adsorption. Modification with a bilaminar coating (electropolymerized *ortho*-phenylenediamine and a fluorinated xerogel) facilitated high selectivity to H_2_S. To demonstrate the biological application of this planar sensor system, H_2_S released from 17β-estradiol-stimulated human umbilical vein endothelial cells (HUVECs) was quantified *in situ* in real-time. Stimulated HUVECs released sustained H_2_S levels for hours before returning to baseline. Cellular viability assays demonstrated negligible cell cytotoxicity at the electrochemical potentials required for analysis.

## Abstract 2

A planar electrode system was developed to permit the real-time, selective detection of hydrogen sulfide (H_2_S) from stimulated cells.

## Graphical abstract

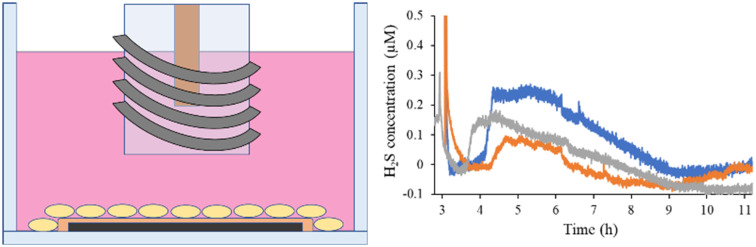

Hydrogen sulfide (H_2_S) was initially perceived to be a hazardous byproduct of decaying organic matter.^1,2^ However, Abe and Kimura^3^ confirmed H_2_S as a biologically important gasotransmitter (*i.e.*, endogenously produced gaseous signaling molecule) alongside nitric oxide (NO) and carbon monoxide (CO).^4^ The near ubiquitous presence of H_2_S within the human body and its now proven roles in the cardiovascular, endocrine, gastrointestinal, and nervous systems have motivated the development of H_2_S donors and analytical methodologies to study its concentration-dependent activity.^5,6^ The earliest detection methods (*e.g.*, methylene blue, sulfide ion selective electrodes) were characterized as having poor detection limits and relied on pH manipulation to shift the dissociation equilibrium.^7–10^ Manipulating pH induced undesirable error (release of acid labile/bound sulfur pools), with overestimation of true biological concentrations (*e.g.*, 1–100 μM).^9,11^ The literature now suggests that H_2_S levels are an order of magnitude lower (100 nM–10 μM),^12–15^ although the concentration range still spans three orders of magnitude. The development of selective, *in situ* detection methods is required to confirm H_2_S's biological concentrations and further elaborate on its physiological roles.

Non-reversible, endpoint detection techniques *via* methylene blue and other fluorophores are still widely used for quantifying cellular-derived H_2_S.^8,14,16,17^ These methodologies limit the ability to observe the dynamic nature of H_2_S production and its subsequent biological application. The ideal analytical methodology for measuring volatile gaseous molecules should enable real-time, *in situ* detection without extensive sample preparation or pH manipulation.^13,15,18^ Electrochemical sensors meet these criteria while also facilitating simple, low-cost fabrication.^19^ Unfortunately, amperometric sensing results in sulfur poisoning, as the elemental sulfur byproduct of the redox reaction passivates electrode surfaces.^20–23^ To date, a number of electrochemical sensor constructs have been reported, often requiring complex coating systems to monitor hydrogen sulfide release from live cells (Table S1†).^24–28^ We have previously developed a robust amperometric H_2_S sensing platform using a surface conditioning procedure on glassy carbon (GC) electrodes.^29^ By exploiting the oxidation-induced sulfur passivation process, electrode surfaces were conditioned until their analytical response to H_2_S stabilized prior to use as H_2_S sensing platforms. Following the subsequent deposition of an electrodeposited *ortho*-phenylenediamine (poly-*o*-PD) permselective coating, the sensors were characterized as being highly selective for H_2_S with low limit of detection (LOD) (<100 nM) over extended continuous use (24 h periods) in proteinaceous media.

The surface-conditioning work described above was carried out using GC macrodisk electrodes suspended in large solution volumes with stirring to homogenize the H_2_S aliquots.^29^ Unfortunately, *in situ* measurements require diffusion to carry H_2_S to the electrode, a process dependent on the distance between the cells and the sensor surface.^30^ Rapid oxidation or scavenging of H_2_S by proteins and metal ions in the media during diffusion are likely to bias the measurements, as suggested by the its short biological lifetime (10 s to 3 min).^4,13^ Previous work in our lab measuring *in situ* NO production showed significant distance-dependence for concentration measurements using suspended electrodes, suggesting the measurement of H_2_S may behave similarly.^30^ Although such error might be mitigated using a microscope (to locate cells) and a micromanipulator, such equipment is costly, bulky, and not amenable to multiplexing or mass production. Herein, we sought to employ a planar H_2_S sensor geometry, wherein cells could be seeded directly upon the electrode surface, reducing the diffusion distance from the source (*i.e.*, cellular production of H_2_S) to the electrode and minimizing potential variability induced by scavenging and oxidation.

Briefly, stencil-printed carbon electrodes (SPCEs) were prepared by applying conductive carbon paste to a plastic substrate using a vinyl stencil. Following a sulfur surface conditioning procedure and application of an optimized H_2_S-selective film (poly-*o*-PD)^29^ to the electrode surface, we compared the analytical merits of the SPCEs to those of GC macrodisk electrodes. A fluorinated-xerogel layer was utilized as a topcoat on the poly-*o*-PD-coated SPCE to provide additional selectivity and prevent degradation of the underlying permselective layer. As a proof-of-concept for *in situ* functionality, the resulting SPCE was used to detect H_2_S released from 17β-estradiol-stimulated human endothelial cells in real-time.

## Experimental

### Materials and instrumentation

Sodium sulfide nonahydrate (Na_2_S·9H_2_O), ethylenediaminetetraacetic acid disodium salt dihydrate (EDTA), *ortho*-phenylenediamine (*o*-PD), sodium nitrite, l-ascorbate, acetaminophen, dopamine hydrochloride, l-cysteine, trimethoxymethylsilane (MTMOS), cysteamine, 17β-estradiol, polyethylene terephthalate (PET) sheets, and fetal bovine serum (FBS) were obtained from Millipore Sigma (Burlington, MA). Ammonium hydroxide, hydrogen peroxide (30 wt%), phenazine methosulfate (PMS), HuMEC media and supplements, trypan blue solution (0.4%), 1X trypsin-EDTA (0.05%), and common laboratory salts were purchased from Thermo Fisher Scientific (Waltham, MA) (heptadecafluoro-1,1,2,2-tetrahydrodecyl)trimethoxysilane (17FTMS) was obtained from Gelest (Morrisville, PA). The 3-(4,5-dimethylthiazol-2-yl)-5-(3-carboxymethoxyphenyl)-2-(4-sulfo-phen-yl)-2*H*-tetrazolium inner salt (MTS) was purchased from Promega (Madison, WI). Nitric oxide (99.5%), argon (99.995%; Ar), and nitrogen (99.998%; N_2_) gases were purchased from Airgas National Welders (Durham, NC). Human umbilical vein endothelial cells (HUVEC; ATCC# CRL 1730) were obtained from the UNC Tissue Culture Facility (Chapel Hill, NC). Carbon ink (CI-2042) was gifted from Engineered Materials Systems, Inc. (Delaware, OH). All other chemicals were reagent grade and used as received.

All water used for solution preparation was purified using a Millipore Milli-Q UV Gradient A10 water purification system (Bedford, MA) to a resistivity of 18.2 MΩ cm and total organic content of ≤6 ppb. Hydrogen sulfide stock solutions were prepared daily by dissolving 12.0 mg of Na_2_S·9H_2_O in 10.0 mL of deoxygenated 150 μM aqueous EDTA. The headspace of the vial containing the stock solution was purged with N_2_ gas and sealed with a rubber septum to prevent oxidation. Stock concentration was determined by iodometric titration.^31^ Saturated NO solution (1.9 mM) was prepared by purging 25 mL of phosphate buffered saline (10 mM, pH 7.4; PBS) with Ar for 25 min, followed by NO gas for 25 min over ice.^32^

Electrochemical experiments were performed using a CH Instruments 1030 eight-channel potentiostat (Austin, TX). The electrochemical cell for H_2_S calibration and selectivity testing was composed of either 3 mm diameter glassy carbon electrodes (CH Instruments; GCE) or custom stencil-printed carbon electrodes (SPCEs), a common silver–silver chloride (Ag|AgCl; 3.0 M KCl; CH Instruments) reference electrode, and a coiled platinum wire counter electrode. All working potentials are *versus* the Ag|AgCl reference electrode. Electrochemical measurements were performed in 20 mL of deoxygenated PBS at room temperature, unless otherwise specified.

### Manufacturing stencil-printed carbon electrodes

Sheets of PET (1 mm thick) were cut into rectangles (15.8 × 25.4 mm or 15.8 × 35 mm) using a laser cutter (Universal Laser Systems; Scottsdale, AZ). Vinyl masks were prepared using 0.003′′ thick adhesive vinyl and cut using a Roland GS-24 Vinyl Cutter (Irvine, CA). Two different electrode designs were employed: dual electrodes (3.2 mm × 19.0 mm per electrode) for analytical performance and selectivity analysis and a single, thinner electrode (1.6 mm × 25.4 mm) for cellular measurements. A depiction of this configuration is provided in ESI† (Fig. S1). The masks were transferred to the PET, and carbon ink was deposited onto the exposed plastic. Excess ink was removed using a razor blade. The ink was cured in an oven at 75 °C for 90 min, after which the masks were removed to reveal the completed electrode. For the dual electrode system, Kapton tape was applied to the electrodes to delineate the lead connections from the working electrode area (3.2 mm × 6.5 mm).

### Electropolymerized *o*-PD film and fluorinated xerogel deposition

Electropolymerized *o*-PD was deposited using cyclic voltammetry (CV) as previously described.^29,33^ Briefly, electrodes were immersed in a 10 mM solution of *o*-PD in PBS. The potential was scanned between 0.0 and +1.0 V at a scan rate of 10 mV s^−1^ for a total of 20 cycles. The coated electrodes were rinsed with water to remove any unbound poly-*o*-PD and allowed to dry. A fluorinated sol (30% v/v 17FTMS, balance MTMOS) was prepared using 4.6 mL ethanol, 924 μL MTMOS, 276 μL 17FTMS, 1280 μL water, and 80 μL 0.5 M HCl.^30^ The solution was stirred vigorously for 1 h prior to using an airbrush gun (Iwata HP-BC1 Plus; Yokohama, Japan) to spray coat the sol onto electrodes. The airbrush gun was positioned 40 cm from the electrode and sprayed for 5 s at 42 psi, constituting one coating. After a 30 min drying period, a second sol coat was applied using the same parameters. The xerogel-coated electrodes were dried for ≥24 h prior to further use.

### Surface preconditioning and calibration procedures

All electrochemical measurements were performed at +0.3 V using constant potential amperometry. As previously detailed,^29^ the surface conditioning procedure deposits sulfur onto the electrode surface until the analytical performance of the sensor stabilizes. This process was performed *via* repeated standard calibrations. We define a standard calibration as five consecutive 20 μL aliquots of the H_2_S stock solution spaced 100 s apart into 20 mL of stirred, deoxygenated PBS. Each electrode tested, whether bare or coated with poly-*o*-PD and/or xerogel, was subjected to at least three standard calibrations prior to use, ensuring stable performance. Following surface conditioning, an additional standard calibration was performed to determine analytical merits, including sensitivity, LOD, and background current.

### Selectivity measurements

Following surface conditioning and analytical characterization, current responses to injections of the following interferents were measured for each electrode: nitrite (1 M), ascorbate (1 M), acetaminophen (50 mM), dopamine (100 mM), H_2_O_2_ (1 M), ammonium (1 M), glutathione (100 mM), cysteine (100 mM), cysteamine (100 mM), and NO (1.9 mM). Of note, the sensor's response to each interferent's responses was tested in fresh, deoxygenated PBS (*i.e.*, without hydrogen sulfide or other interferents present) to definitively isolate their individual current contributions. Electrode sensitivity to each interferent was calculated and compared to the H_2_S sensitivity to determine the selectivity coefficient (*k*) using eqn (1):1
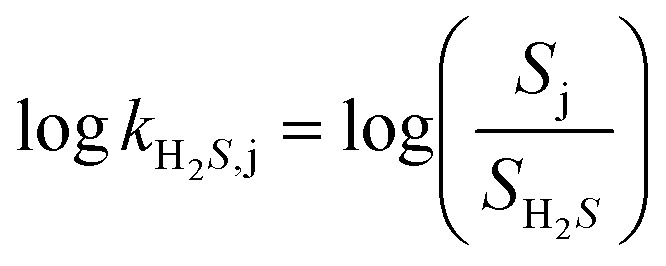
where *S*_H_2_S_ and *S*_j_ represent the sensitivities calculated for H_2_S and interferent j, respectively.

### Measurement of estradiol-induced H_2_S release from HUVECs

**Fig. 1:**
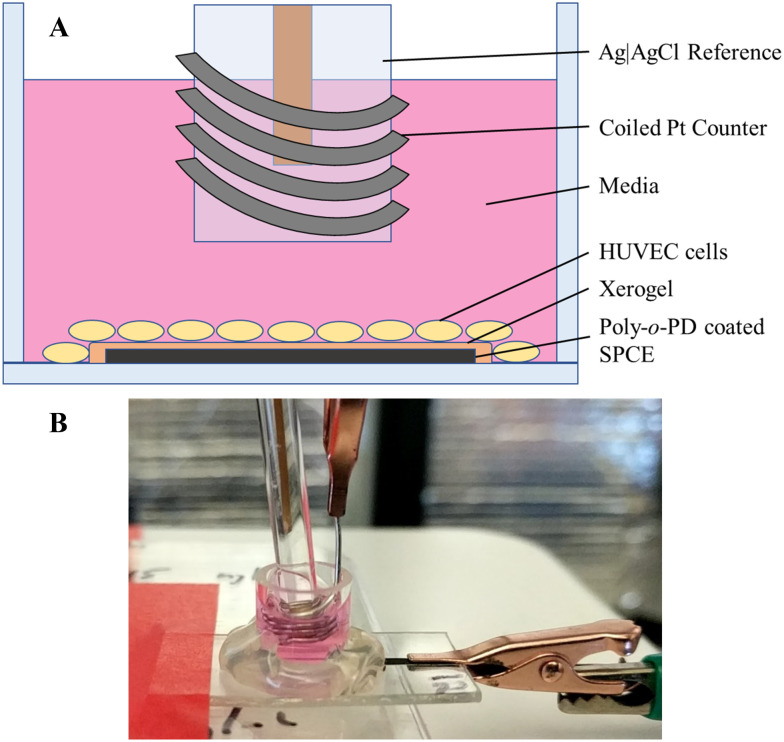
Set-up for the *in situ* measurement of 17β-estradiol-induced H_2_S release from HUVEC cells. (A) Cut-out side view of the well atop a coated SPCE. HUVEC cells are seeded upon the electrode and surrounding PET. A Ag|AgCl reference electrode and coiled Pt counter electrode are submerged in the media. (B) Image of the completed experimental set-up.

The HUVECs were grown in HuMEC media combined with a HuMEC supplement mix (5 mL), bovine pituitary extract (25 mg), and FBS (5% v/v) and stored in a humidified incubator (37 °C, 5% v/v CO_2_). The coated and preconditioned electrodes had glass cell cloning cylinders (10 × 10 mm) adhered to the PET surface to form a well for cell seeding (Fig. 1). Sensitivity of the exposed electrode within the cylinder was determined using a standard calibration. The electrode was rinsed copiously with water and allowed to dry. Cells (100 000 cells per mL) were seeded by adding 150 μL of the cell solution to each well and allowing them to adhere for 3 h in the incubator. Following adherence to the electrode surface, the media was removed and replaced with 300 μL of fresh, prewarmed media. The electrodes were connected to the potentiostat (+0.3 V working potential), and Ag|AgCl reference and coiled Pt counter electrodes were inserted into each well (Fig. 1). After a 3 h polarization, a 1.5 μL bolus injection of 2 μM 17β-estradiol in EtOH was added, resulting in a 10 nM estradiol solution. The estradiol-induced release of H_2_S was then monitored for approximately 12 h.

### Cellular viability assays

Cellular viability was assessed following exposure to 17β-estradiol using two separate methods: MTS assay and trypan blue staining. The MTS assay is a determination of metabolic activity *via* the reduction of a colorimetric dye. After 12 h, the media containing estradiol was removed and the cells were washed with 300 μL sterile PBS. Cells were then incubated with 300 μL of a HuMEC/MTS/PMS mixture (105/20/1, v/v/v) for 3 h at 37 °C. Aliquots of the MTS solution (100 μL) were transferred to a 96-well plate with quantification by absorbance measurement at 490 nm using a SpectraMax M2e microplate spectrophotometer (Molecular Devices, Sunnyvale, CA). The cell viability was calculated as follows:2
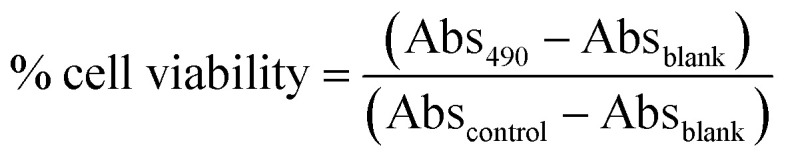
The controls (Abs_control_) consisted of cells seeded in a glass cell cloning well adhered to bare PET (*i.e.*, no SPCE or coatings). These cells were not exposed to estradiol, electrode coatings, or the working potential, and were stored in the incubator throughout the test. The blanks (Abs_blank_), originally treated in the same fashion as the controls, were fixed with 300 μL ethanol (70%, 1 h) prior to MTS. Both blank and control wells were treated with 300 μL of the HuMEC/MTS/PMS mixture under the conditions detailed above.

As trypan blue analysis requires cells to be removed from the electrode surface, 50 μL of trypsin-EDTA (0.05%) was added to the wells and allowed to incubate for 10 min. Media (50 μL) was then added to the free-floating cells to inhibit the trypsin activity. A 1 : 1 mixture of the cell solution and trypan blue solution was prepared and mixed immediately before analysis. The solution was then added to a hemocytometer for counting on an optical microscope. Of note, dead cells were denoted by their blue coloration as the trypan blue dye is itself impermeable to intact (*i.e.*, live) cell membranes. Cell counts were performed 4 times per well. Counting of positive (live) controls were performed under the same conditions as the MTS assay controls.

### Data analysis

Values for sensitivity, limit of detection, selectivity, and cellular viability are expressed as the mean ± the standard deviation. Comparisons between data sets were performed using a two-tailed Student's *t*-test with *p* < 0.05 indicating statistical significance.

## Results and discussion

Glassy carbon electrodes are often comprised of GC disks embedded within an insulator (*i.e.*, plastic or glass) to create a planar surface. While these electrodes have been used in previous microfluidic devices, the configuration is bulky and limits translation into existing platforms or point-of-care applications.^34^ As a simple and inexpensive alternative, stencil-printed carbon electrodes (SPCEs) were chosen as the most efficient and reproducible methodology for developing a planar carbon electrode system.^35,36^ By applying a curable, conductive carbon paste to the substrate using a mask, the SPCEs allow for the production of electrodes with highly-controllable and customizable dimensions. Many research groups have developed SPCEs for paper-based point-of-care devices due to their low weight, planar design, and reasonable electroconductivity.^35,37–39^ The use of H_2_S-selective coatings enables the design of a planar H_2_S sensor capable of real-time *in situ* H_2_S measurement from cells and tissues or integration into existing platforms, such as microfluidic devices.

### Characterization of bare and poly-*o*-PD-modified SPCEs

**Fig. 2:**
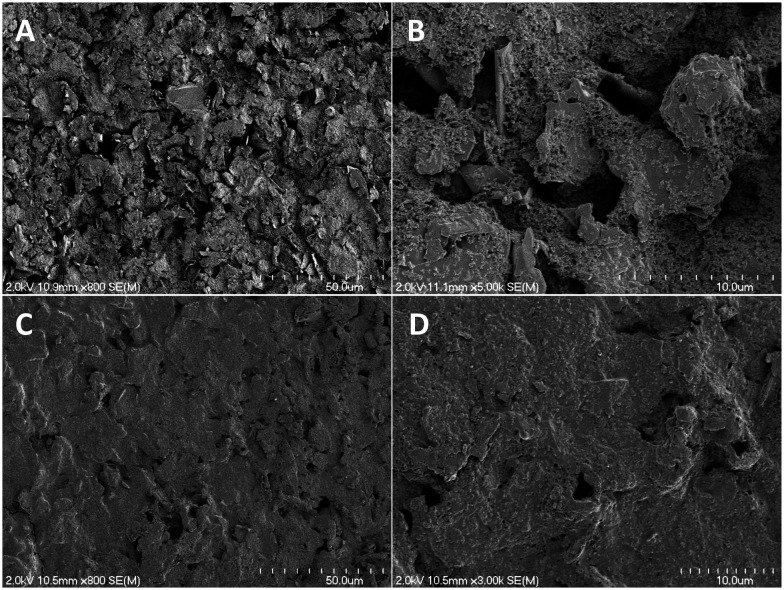
Representative SEM images of the SPCE surface with and without the xerogel layer. (A) Bare SPCEs surface with (B) a magnified image for added detail regarding the granular binder. (C) Image of SPCE surface with the addition of two xerogel coatings and (D) a magnified portion depicting the notable xerogel coverage.

The SPCEs were prepared on PET sheets as carbon inks are primarily manufactured for use on paper, plastic, or ceramic substrates.^35,36^ Vinyl masks were applied to the PET. Conductive carbon ink was stenciled onto the plastic surface. The electrode was then cured in an oven (75 °C) for 90 min. Following curing, the mask was removed to expose the completed SPCE. A piece of Kapton tape was applied to delineate the working electrode (3.2 mm × 6.5 mm) from the connecting leads (Fig. S1†). The SPCE's appearance and thickness were characterized by scanning electron microscopy (SEM) (Fig. 2A and B). The electrode surface was particularly rough and consisted of carbon shards held together with a granular-looking polymer binder.^36^ Cross-sectional SEM images indicated approximate thickness of the cured carbon ink to be 15.5 ± 1.1 μm, roughly 5 times thinner than the vinyl mask (∼76 μm).

Bare SPCEs were modified with permselective poly-*o*-PD film *via* CV.^29^ The electropolymerization process generates cationic radical monomers *via* oxidation, which couple together to form oligomer chains. The oligomers eventually grow beyond their solubility limit and precipitate out of solution onto the electrode surface, forming a permselective film. The cyclic voltammogram for electropolymerization on SPCE closely matches the major features observed on GC electrodes, with an anodic peak at +0.4 V and a broad shoulder spanning +0.5 to +0.8 V (Fig. S2†). Self-terminating film growth was confirmed by passivation of current following the initial potential sweep. The approximately three-fold increase in peak current in SPEC *versus* the GC disk is attributed to greater geometric surface area (20.15 and 7.07 mm^2^, respectively). The matching CV features and autopassivation verify the poly-*o*-PD film deposition on the SPCE.

**Fig. 3:**
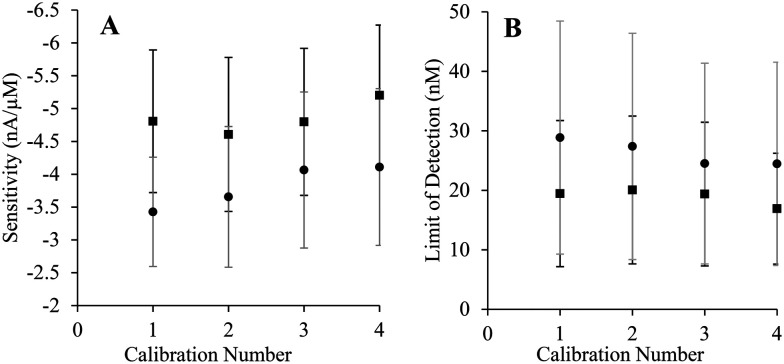
Analytical performance of bare (squares) and poly-*o*-PD-coated (circles) SPCEs as a function of the number of standard calibrations performed. (A) Sensitivity of the electrodes. (B) Limit of detection. All working potentials were +0.3 V (*N* = 4). Error bars for poly-*o*-PD-coated SPCEs are provided in grey color. Calibration curves are available in ESI† (Fig. S3).

Consecutive standard H_2_S calibrations were performed with both bare and poly-*o*-PD-modified SPCEs to assess compatibility with the surface conditioning procedure.^29^ Neither the H_2_S sensitivity nor the LOD was changed significantly over four calibrations (Fig. 3), indicating successful surface conditioning. Furthermore, subsequent exposure to the elemental sulfur byproduct did not result in performance variation. The surface conditioning protocol was thus employed for all sensor fabrication hereafter.

The analytical merits of the preconditioned SPCE (*i.e.*, sensitivity and LOD) were compared to those of GC electrodes. As shown in Table 1, bare SPCEs exhibited similar performance to GC electrodes. However, GC electrodes exhibited significantly greater sensitivity upon normalization of the electrodes' geometric surface area. Logically, it was anticipated that the GC electrodes would outperform the SPCEs, as GC is a commonly employed electrode material with excellent conductivity and resistance properties.^40,41^ The lower sensitivity per normalized area for SPCE also corresponds with slightly elevated LODs, although they are still well below that required for biological experiments (∼100 nM). A significant difference between the two electrode materials was evident upon coating with the permselective film. The GC electrode was heavily passivated in the presence of the film, whereas the SPCEs' sensitivity only diminished slightly. This difference in passivation may be attributed to surface topography (Fig. 2A and B). Polished GC presents a “pristine” flat surface where *o*-PD oligomers pack together tightly, inhibiting H_2_S diffusion to a large portion of the electrode interface and reducing sensitivity. The rough, uneven surface characteristic of the SPCE does not allow for such packing. In turn, a more disordered packing would lead to less passivation as the film coverage is less dense (Table 1).

**Table 1:** Analytical merits for bare *versus* poly-*o*-PD-coated glassy carbon and stencil-printed carbon electrodes for the oxidation of hydrogen sulfide in PBS

Electrode (coating)	Raw sensitivity^a^ (nA μM^−1^)	LOD^b^ (nM)	Normalized sensitivity^c^ (nA μM^−1^ mm^−2^)
GC (bare)	−4.03 ± 0.91	1 ± 0	−0.57 ± 0.13
GC (*o*-PD)	−0.28 ± 0.06	15 ± 12	−0.04 ± 0.01
SPCE (bare)	−5.20 ± 1.07	16 ± 9	−0.26 ± 0.05
SPCE (*o*-PD)	−4.11 ± 1.19	24 ± 17	−0.20 ± 0.06

a Sensitivity calculated using a standard calibration following surface conditioning.

b S/N = 3.

c Sensitivity was normalized per mm^2^ using the individual geometric area: 7.07 mm^2^ for GCE and 20.15 mm^2^ for SPCE.

The packing phenomenon at the electrode surface was supported upon determination of the selectivity of bare and film-modified GC electrodes and SPCEs to H_2_S over potential interferents (Table 2). Selectivity coefficients, determined using eqn (1), were similar between the bare versions of each electrode material, as might be expected. Upon addition of the poly-*o*-PD film, the SPCEs were significantly more susceptible to interferent oxidation than their GC counterparts, particularly for low oxidation potential interferents (*i.e.*, ascorbate, acetaminophen, dopamine, and cysteine).^29^ As the presence of larger (*i.e.*, greater molecular weight) interferents (*e.g.*, dopamine and cysteine) resulted in little to no increase in selectivity to H_2_S, it was confirmed that the size-exclusion properties of the film for SPCEs were in fact compromised by the inefficient poly-*o*-PD oligomer packing.

**Table 2:** Selectivity of bare and poly-*o*-PD coated glassy carbon and stencil-printed carbon electrodes over common biological interferents

Electrode (coating)	Selectivity coefficient^a^					
	AA	AP	DA	CYS	H_2_O_2_	NO_2_^−^
GCE (bare)	−0.04 ± 0.11	−1.89 ± 0.14	0.76 ± 0.18	−1.77 ± 0.14	−4.58 ± 0.30	<−5^b^
GCE (*o*-PD)	−2.39 ± 0.16	−4.49 ± 0.42	−2.50 ± 0.21	−2.78 ± 0.16	−5.01 ± 0.15	<−5^b^
SPCE (bare)	0.18 ± 0.11	−1.45 ± 0.08	0.91 ± 0.10	−1.59 ± 0.10	−4.50 ± 0.34	<−5^b^
SPCE (*o*-PD)	−0.89 ± 0.08	−2.56 ± 0.25	0.31 ± 0.38	−2.04 ± 0.16	−4.92 ± 0.09	−4.33 ± 0.06

a Selectivity coefficient calculated using eqn (1) against the following interferents: l-ascorbate (AA), acetaminophen (AP), dopamine (DA), cysteine (CYS), hydrogen peroxide (H_2_O_2_), and nitrite (NO_2_^−^).

b No current change was observed upon interferent addition, so theoretically the selectivity coefficient is <−5.

### Analytical performance of xerogel-coated SPCEs

Fluorinated xerogels have previously been employed as an external electrode barrier to facilitate the fabrication of NO sensors.^30,42,43^ The xerogel enables greater NO selectivity by providing a hydrophobic and size-exclusion permselective coating that also protects the electrode surface from undesirable chemical reactions and degradation.^30,42,43^ Of note, H_2_S is uncharged and may be thought of as lipophilic, thus allowing it to more easily transverse the hydrophobic xerogel coating while larger and/or charged species are essentially rejected.^4^ To address the reduced effects of the poly-*o*-PD film on SPCE due to inefficient packing, a xerogel coating was applied on top of the poly-*o*-PD film to enhance selectivity for H_2_S over anticipated biological interferents. As depicted in the SEM images, the additional xerogel layer also altered the microscopic topography (Fig. 2C and D). Indeed, the xerogel created a more uniform coating and electrode. As anticipated, a decrease in the sensitivity and concomitant increase in LOD were observed following the application of the first xerogel layer (Table 3). The addition of a second xerogel coat resulted in only minor improvements in sensor performance (*e.g.*, sensitivity, selectivity), suggesting that the initial coating was sufficient.

**Table 3:** Analytical performance of stencil-printed carbon electrodes modified with both poly-*o*-PD films and fluorinated xerogel topcoats

SPCE coating	Sensitivity (nA μM^−1^)	LOD^b^ (nM)	Selectivity coefficient^a^						
			AA	AP	DA	CYS	H_2_O_2_	NO_2_^−^	Ammonium
Bare	−5.20 ± 1.07	16 ± 9	0.18 ± 0.11	−1.45 ± 0.08	0.91 ± 0.10	−1.59 ± 0.10	−4.50 ± 0.34	−5.64 ± 0.23	−2.77 ± 0.11
*o*-PD	−4.11 ± 1.19	24 ± 17	−0.89 ± 0.08	−2.56 ± 0.25	0.31 ± 0.38	−2.04 ± 0.16	−4.92 ± 0.09	−4.33 ± 0.06	−2.55 ± 0.13
*o*-PD/1x XG	−1.87 ± 0.19	69 ± 24	−2.43 ± 0.40	−3.21 ± 0.12	−0.96 ± 0.09	−2.57 ± 0.07	−4.63 ± 0.14	−3.64 ± 0.04	−2.75 ± 0.03
*o*-PD/2x XG^c^	−1.71 ± 0.36	80 ± 20	−2.66 ± 0.44	−3.60 ± 0.29	−1.32 ± 0.22	−2.72 ± 0.25	−4.70 ± 0.20	−3.65 ± 0.04	−2.74 ± 0.03

a Selectivity coefficient calculated using eqn (1) against the following interferents: l-ascorbate (AA), acetaminophen (AP), dopamine (DA), cysteine (CYS), hydrogen peroxide (H_2_O_2_), nitrite (NO_2_^−^), and ammonium.

b S/N = 3.

c Two xerogel topcoats were applied with a 30 min dry time between spray coatings.

Further demonstrating the advantages of utilizing a xerogel topcoat was the significant increase in selectivity to H_2_S over the most problematic large interferents, including ascorbate, acetaminophen, dopamine, and cysteine (Table 3). This benefit correlates directly to the known size-exclusion properties of the fluorinated xerogel.^30,42,43^ Oddly, the selectivity coefficient over nitrite diminished, although the sensor remained highly selective for H_2_S over this anion. Brown *et al.* reported that poly-*o*-PD film may retain a net positive charge.^29^ As such, the xerogel application may slightly attract the negatively-charged nitrite. Moreover, the selectivity over NO was also lower than expected (−2.55 ± 0.11). As an equally lipophilic molecule, NO may easily penetrate the xerogel. Any selectivity would thus be solely imparted by the relatively low applied potential (*i.e.*, +0.3 V). These results also suggest that the carbon ink common to SPCEs may allow for nitrite and NO oxidation at lower potentials than GC, thus altering the selectivity for H_2_S. The selectivity coefficients for NO and nitrite (−2.55 ± 0.11 and −3.65 ± 0.04, respectively) indicate, while slightly lower than expected, the sensor is still highly selective toward hydrogen sulfide with the xerogel coatings. Finally, the H_2_S selectivity of the xerogel-coated SPCE sensors over other sulfur-containing biological interferents was comparable to that for the poly-*o*-PD-modified GC electrodes (*e.g.*, −3.46 ± 0.11 and −1.88 ± 0.12 against glutathione and cysteamine, respectively).^29^

Overall, the addition of the xerogel topcoats improves selectivity over crucial biological interferents and maintains sufficient sensitivities with LODs below 100 nM and rapid response times (16.8 ± 7.4 s; 95% max Δ*i*). As two xerogel coatings assures full surface coverage without negatively impacting performance, the bilaminar combination of poly-*o*-PD and two xerogel applications was selected for all subsequent work.

### *In situ* detection of 17β-estradiol-induced H_2_S release

**Fig. 4:**
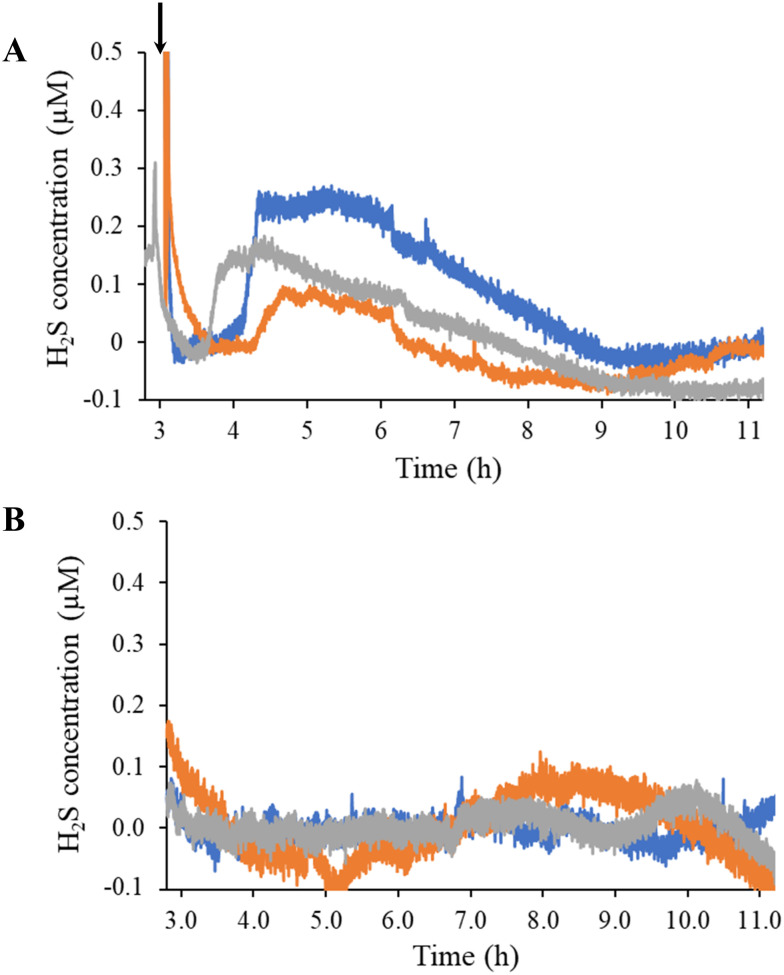
Hydrogen sulfide-release profiles of (A) 17β-estradiol-stimulated and (B) unstimulated HUVECs measured using a SPCE modified with poly-*o*-PD film and two xerogel coats. Each color trace correlates to a separate, independent sensor. Newly prepared sensors were used in each experiment. Polarization background was subtracted out. Bolus injection of 17β-estradiol is indicated by the arrow. Total 17β-estradiol concentration was 10 nM.

Estrogen levels are known to have a direct effect on H_2_S levels in both the cardiovascular and skeletal system.^44–48^ As the predominant estrogen hormone, 17β-estradiol stimulates the activity of cystathionine γ-lyase (CSE), a primary H_2_S-producing enzyme in cells (*e.g.*, HUVECs) and vascular tissue.^45,48^ To demonstrate the sensor's utility for real-time, *in situ* H_2_S monitoring, HUVECs were seeded directly onto the coated electrode surface and stimulated with a bolus injection of 17β-estradiol (Fig. 1). Of note, the injection prompted a spike in current originating from opening the Faraday cage containing the electrodes and disturbing the stationary media with the injection (Fig. 4). The background current eventually stabilized with the first detectable signal due to H_2_S observed approximately 1 h post injection. The H_2_S levels plateaued to 100–250 nM levels and remained constant for up to 3 h before gradually returning to baseline. For all trials, H_2_S was observed to be release for a total of 4–5 h after the initial detection.

The overall response dynamics observed (*e.g.*, sharp rise and gradual return to baseline) were expected based on previous literature, although the time frame and magnitude proved markedly different.^45,48^ We postulated that the disparities in peak concentration (100 to 250 nM compared to 1 to 2 μM in the literature^45,48^) results from the number of HUVECs used to generate the H_2_S. Zhou *et al.* do not state a detailed seeding protocol (*i.e.*, number of cells).^48^ Liu *et al.* reported that the number of HUVECs stimulated impacts the peak concentration and release duration of NO.^49^ Longer times prior to initial H_2_S detection and extended release durations could also be attributed to the bolus injection methodology, which requires diffusion to transport 17β-estradiol to the cells over a protracted period instead of instantly accessing cells, as would be the case if the entire solution was immediately homogenous. Starting with a homogenous solution of 17β-estradiol to counteract this diffusion problem resulted in an immediate spike in current with no stabilization of background, thus convoluting H_2_S quantification.

Following overnight (∼18 h) of continuous measurement (at the applied potential), HUVEC viability was assessed using MTS and trypan blue assays. Cell viability is an important parameter to measure as applied potential may induce cell lysis through membrane polarization.^50,51^ The MTS assay indicated ∼78% active cells compared to adsorbed cells with no applied potential. Likewise, the percentage of intact cells following applied potential exposure as determined using trypan blue was found to be ∼72%, analogous to control cells without applied potential. Together, these two assays that nearly three-quarters of the cells maintain their viability for at least 18 hours at +0.3 V when seeded on top of an electrode in this sensor configuration.

## Conclusions

Planar SPCEs were developed and characterized for their potential to facilitate real-time, selective detection of H_2_S. A bilaminar coating consisting of a poly-*o*-PD electrodeposited film and xerogel topcoats was found to be highly selective towards H_2_S with sufficient sensitivity (−1.7 ± 0.4 nA μM^−1^) and biologically-relevant LODs (80 ± 20 nM). As a proof-of-concept of biological utility, HUVECs were seeded directly onto the surface of the SPCE and stimulated with 17β-estradiol, leading to the evolution of H_2_S from the cells. The H_2_S levels peaked at ∼1 h post 17β-estradiol injection, and sustained release was measured at near constant levels thereafter for several hours before returning to baseline. Cell viability assays demonstrated that neither the bilaminar coating nor the applied potential influenced cellular metabolism or membrane integrity relative to controls. The SPCE-based sensor indicates potential for real-time measurement of cell-derived H_2_S. This system has the potential to be applied to additional cell lines and/or H_2_S stimulants/inhibitors for further characterization of H_2_S signaling/generation. Moreover, the planar nature of the sensor may enable integration into microfluidic or point-of-care devices for use a compact, sensitive diagnostic tool.

## Author contributions

J. R. Hall contributed to the research on conceptualization, primary investigator, data analysis and visualization, and writing the original draft, review, and editing. J. B. Taylor contributed on methodology of cell growth and cell viability assays and article writing – review and editing. T. M. Bradshaw contributed as an investigator (SEM imaging) and article writing – review and editing. M. H. Schoenfisch contributed with funding acquisition, project supervision, and article writing – review and editing.

## Conflicts of interest

None.

## Supplementary Material

SD-002-D2SD00179A-s001

## References

[cit1] Kolluru G. K., Shen X., Kevil C. G. (2013). A Tale of Two Gases: NO and H2S, Foes or Friends for Life?. Redox Biol..

[cit2] Szabo C. (2018). A Timeline of Hydrogen Sulfide (H2S) Research: From Environmental Toxin to Biological Mediator. Biochem. Pharmacol..

[cit3] Abe K., Kimura H. (1996). The Possible Role of Hydrogen Sulfide as an Endogenous Neuromodulator. J. Neurosci..

[cit4] Wang R. (2002). Two's Company, Three's a Crowd: Can H2S Be the Third Endogenous Gaseous Transmitter?. FASEB J..

[cit5] Tinajero-Trejo M., Jesse H. E., Poole R. K. (2013). Gasotransmitters, Poisons, and Antimicrobials: It's a Gas, Gas, Gas!. F1000Prime Rep..

[cit6] Li L., Hsu A., Moore P. K. (2009). Actions and Interactions of Nitric Oxide, Carbon Monoxide and Hydrogen Sulphide in the Cardiovascular System and in Inflammation - a Tale of Three Gases!. Pharmacol. Ther..

[cit7] Fogo J., Popowsky M. (1949). Spectrophotometric Determination of Hydrogen Sulfide. Anal. Chem..

[cit8] Kolluru G. K., Shen X., Bir S. C., Kevil C. G. (2013). Hydrogen Sulfide Chemical Biology: Pathophysiological Roles and Detection. Nitric Oxide.

[cit9] Lawrence N. S., Davis J., Compton R. G. (2000). Analytical Strategies for the Detection of Sulfide: A Review. Talanta.

[cit10] Garcia-Calzada M., Marban G., Fuertes A. B. (1999). Potentiometric Determination of Sulphur in Solid Sampleswith a Sulphide Selective Electrode. Anal. Chim. Acta.

[cit11] Kraus D. W. , Doeller J. E. and Zhang X., Electrochemical Sensors for the Determination of Hydrogen Sulfide Production in Biological Samples, in Electrochemical Sensors, Biosensors and their Biomedical Applications, 2008, pp. 213–235

[cit12] Wallace J. L., Wang R. (2015). Hydrogen Sulfide-Based Therapeutics: Exploiting a Unique but Ubiquitous Gasotransmitter. Nat. Rev. Drug Discovery.

[cit13] Whitfield N. L., Kreimier E. L., Verdial F. C., Skovgaard N., Olson K. R. (2008). Reappraisal of H2S/Sulfide Concentration in Vertebrate Blood and Its Potential Significance in Ischemic Preconditioning and Vascular Signaling. Am. J. Physiol..

[cit14] Shen X., Pattillo C. B., Pardue S., Bir S. C., Wang R., Kevil C. G. (2011). Measurement of Plasma Hydrogen Sulfide in Vivo and in Vitro. Free Radical Biol. Med..

[cit15] Furne J., Saeed A., Levitt M. D. (2008). Whole Tissue Hydrogen Sulfide Concentrations Are Orders of Magnitude Lower than Presently Accepted Values. Am. J. Physiol..

[cit16] Peng B., Chen W., Liu C., Rosser E. W., Pacheco A., Zhao Y., Aguilar H. C., Xian M. (2014). Nucleophilic Substitution-Cyclization Based Fluorescent Probes for Hydrogen Sulfide Detection and Bioimaging. Chem. – Eur. J..

[cit17] Lin V. S., Lippert A. R., Chang C. J. (2013). Cell-Trappable Fluorescent Probes for Endogenous Hydrogen Sulfide Signaling and Imaging H2O2-Dependent H2S Production. Proc. Natl. Acad. Sci. U. S. A..

[cit18] Olson K. R. (2009). Is Hydrogen Sulfide a Circulating “Gasotransmitter” in Vertebrate Blood?. Biochim. Biophys. Acta, Bioenerg..

[cit19] Shah S. S., Aziz M. A., Oyama M., Al-Betar A. R. F. (2021). Controlled-Potential-Based Electrochemical Sulfide Sensors: A Review. Chem. Rec..

[cit20] Hall J. R., Schoenfisch M. H. (2018). Direct Electrochemical Sensing of Hydrogen Sulfide without Sulfur Poisoning. Anal. Chem..

[cit21] Szynkarczuk J., Komorowski P. G., Donini J. C. (1994). Redox Reactions of Hydrosulphide Ions on the Platinum Electrode - I. The Presence of Intermediate Polysulphide Ions and Sulphur Layers. Electrochim. Acta.

[cit22] Kapusta S., Viehbeck A., Wilhelm S. M., Hackerman N. (1983). The Anodic Oxidation of Sulfide on Platinum Electrodes. J. Electroanal. Chem. Interfacial Electrochem..

[cit23] Vandeberg P. J., Kowagoe J. L., Johnson D. C. (1992). Pulsed Amperometric Detection of Sulfur Compounds: Thiourea at Gold Electrodes. Anal. Chim. Acta.

[cit24] Hu X. B., Liu Y. L., Zhang H. W., Xiao C., Qin Y., Duo H. H., Xu J. Q., Guo S., Pang D. W., Huang W. H. (2016). Electrochemical Monitoring of Hydrogen Sulfide Release from Single Cells. ChemElectroChem.

[cit25] Asif M., Aziz A., Wang Z., Ashraf G., Wang J., Luo H., Chen X., Xiao F., Liu H. (2019). Hierarchical CNTs@CuMn Layered Double Hydroxide Nanohybrid with Enhanced Electrochemical Performance in H2S Detection from Live Cells. Anal. Chem..

[cit26] Jeromiyas N., Mani V., Chang P. C., Huang C. H., Salama K. N., Huang S. T. (2021). Anti-Poisoning Electrode for Real-Time in-Situ Monitoring of Hydrogen Sulfide Release. Sens. Actuators, B.

[cit27] Panda A. K., Keerthi M., Sakthivel R., Dhawan U., Liu X., Chung R. J. (2022). Biocompatible Electrochemical Sensor Based on Platinum-Nickel Alloy Nanoparticles for In Situ Monitoring of Hydrogen Sulfide in Breast Cancer Cells. Nanomaterials.

[cit28] Gu W., Zheng W., Liu H., Zhao Y. (2021). Electroactive Cu2O Nanocubes Engineered Electrochemical Sensor for H2S Detection. Anal. Chim. Acta.

[cit29] Brown M. D., Hall J. R., Schoenfisch M. H. (2019). A Direct and Selective Electrochemical Hydrogen Sulfide Sensor. Anal. Chim. Acta.

[cit30] Brown M. D., Schoenfisch M. H. (2019). Selective and Sensocompatible Electrochemical Nitric Oxide Sensor with a Bilaminar Design. ACS Sens..

[cit31] Pawlak Z., Pawlak A. S. (1999). Modification of Iodometric Determination of Total and Reactive Sulfide in Environmental Samples. Talanta.

[cit32] Park S. S., Kim J., Lee Y. (2012). Improved Electrochemical Microsensor for the Real-Time Simultaneous Analysis of Endogenous Nitric Oxide and Carbon Monoxide Generation. Anal. Chem..

[cit33] Brown M. D., Schoenfisch M. H. (2016). Nitric Oxide Permselectivity in Electropolymerized Films for Sensing Applications. ACS Sens..

[cit34] Walton E. L., Mcnamara S., Scott R., Spence D. M. (2014). 3D Printed Microfluidic Devices with Integrated Versatile and Reusable Electrodes. Lab Chip.

[cit35] Oh J., Chow K. (2015). Recent Developments in Electrochemical Paper-Based Analytical Devices. Anal. Methods.

[cit36] Wang J., Tian B., Nascimento B., Angnes L. (1998). Performance of Screen-Printed Carbon Electrodes Fabricated from Different Carbon Inks. Electrochim. Acta.

[cit37] Liu H., Xiang Y., Lu Y., Crooks R. M. (2012). Aptamer-Based Origami Paper Analytical Device for Electrochemical Detection of Adenosine. Angew. Chem., Int. Ed..

[cit38] Cunningham J. C., Kogan M. R., Tsai Y., Luo L., Richards I., Crooks R. M. (2016). Paper-Based Sensor for Electrochemical Detection of Silver Nanoparticle Labels by Galvanic Exchange. ACS Sens..

[cit39] Fosdick S. E., Anderson M.
J., Renault C., Degregory P. R., Loussaert J. A., Crooks R. M. (2014). Wire, Mesh, and Fiber Electrodes for Paper-Based Electroanalytical Devices. Anal. Chem..

[cit40] Gross M., Jordan J. (1984). Voltammetry At Glassy Carbon Electrodes. Pure Appl. Chem..

[cit41] Elgrishi N., Rountree K. J., Mccarthy B. D., Rountree E. S., Eisenhart T. T., Dempsey J. L. (2018). A Practical Beginner's Guide to Cyclic Voltammetry. J. Chem. Educ..

[cit42] Jae H. S., Privett B. J., Kita J. M., Wightman R. M., Schoenfisch M. H. (2008). Fluorinated Xerogel-Derived Microelectrodes for Amperometric Nitric Oxide Sensing. Anal. Chem..

[cit43] Hunter R. A., Privett B. J., Henley W. H., Breed E. R., Liang Z., Mittal R., Yoseph B. P., McDunn J. E., Burd E. M., Coopersmith C. M. (2013). *et al.*, Microfluidic Amperometric Sensor for Analysis of Nitric Oxide in Whole Blood. Anal. Chem..

[cit44] Grassi F., Tyagi A. M., Calvert J. W., Gambari L., Walker L. D., Yu M., Robinson J., Li J., Lisignoli G., Vaccaro C. (2016). *et al.*, Hydrogen Sulfide Is a Novel Regulator of Bone Formation Implicated in the Bone Loss Induced by Estrogen Deficiency. J. Bone Miner. Res..

[cit45] Xu X., Yan Q., Liu X., Li P., Li X., Chen Y., Simoncini T., Liu J., Zhu D., Fu X. (2019). 17β-Estradiol Non-Genomically Induces Vascular Endothelial H2S Release by Promoting Phosphorylation of Cystathionine γ-Lyase. J. Biol. Chem..

[cit46] Li H., Mani S., Wu L., Fu M., Shuang T., Xu C., Wang R. (2017). The Interaction of Estrogen and CSE/H2S Pathway in the Development of Atherosclerosis. Am. J. Physiol..

[cit47] Li H., Mani S., Cao W., Yang G., Lai C., Wu L., Wang R. (2012). Interaction of Hydrogen Sulfide and Estrogen on the Proliferation of Vascular Smooth Muscle Cells. PLoS One.

[cit48] Zhou K., Gao Q., Zheng S., Pan S., Li P., Suo K., Simoncini T., Wang T., Fu X. (2013). 17β-Estradiol Induces Vasorelaxation by Stimulating Endothelial Hydrogen Sulfide Release. Mol. Hum. Reprod..

[cit49] Liu Y., Wang X., Xu J., Xiao C., Liu Y., Zhang X., Liu J., Huang W. (2015). Functionalized Graphene-Based Biomimetic Microsensor Interfacing with Living Cells to Sensitively Monitor Nitric Oxide Release. Chem. Sci..

[cit50] Yaoita M., Ikariyama Y., Aizawa M. (1990). Electrical Effects on the Proliferation of Living HeLa Cells Cultured on Optically Transparent Electrode Surface. J. Biotechnol..

[cit51] Oni J., Pailleret A., Isik S., Diab N., Radtke I., Blöchl A., Jackson M., Bedioui F., Schuhmann W. (2004). Functionalised Electrode Array for the Detection of Nitric Oxide Released by Endothelial Cells Using Different NO-Sensing Chemistries. Anal. Bioanal. Chem..

