# Supplementary material for: Planar carbon electrodes for real-time quantification of hydrogen sulfide release from cells
Source: Sens Diagn. 2022 Dec 1;2(1):203–11. doi: 10.1039/d2sd00179a (PMC9850357; doi:10.1039/d2sd00179a)
Supplement: SD-002-D2SD00179A-s001 [file SD-002-D2SD00179A-s001.pdf]

Electronic Supplementary Material (ESI) for Sensors & Diagnostics.
This journal is © The Royal Society of Chemistry 2022

Supporting Information

Planar carbon electrodes for real-time quantification of hydrogen sulfide release from cells

Jackson R. Hall[a], James B. Taylor[a], Taron M. Bradshaw[a], Mark H. Schoenfisch[a,b]*

[a] Department of Chemistry, The University of North Carolina at Chapel Hill, Chapel Hill, North Carolina 27599, United States

[b] Division of Pharmacoengineering and Molecular Pharmaceutics, UNC Eshelman School of Pharmacy, Chapel Hill, NC 27599, United States

*Email: schoenfisch@unc.edu

Table of Contents

1. Table of electrochemical hydrogen sulfide sensors used to monitor live cells — Table S-1

2. Mask schemes for the two SPCE designs — Figure S-1

3. Cyclic voltammograms of *o*-PD upon GC electrodes and SPCEs — Figure S-2

4. Repeated standard calibration curves on bare and *o*-PD coated SPCEs — Figure S-3

Table S-1. Comparison of various, recent hydrogen sulfide electrochemical sensors used to detect hydrogen sulfide release from live cells.

Electrode Material	Method	Limit of Detection	Sensitivity	Response Time	Cell type	Reference
SPCE (*o*-PD/2x XG)	Amperometry	80 nM	1.71 nA/µM	16.8±7.4s	HUVEC	This work
PEDOT-modified nanoporous gold microelectrode	Amperometry	0.1 µM	0.125 nA/µM	1s	HeLa	[1]
LDH wrapped carbon nanotubes	Amperometry	0.3 nM	73 µA/mM cm^{-2}	3s	Sulfate reducing bacteria; Melanoma (A375)	[2]
Reduced graphene oxide-MoS_2	Amperometry	10 nM	0.0008 µA/µM	<5s	E. coli	[3]
PtNi alloy nanoparticles	Amperometry	0.004 µM	0.323 µA/µM cm^{-2}	4.8s	Breast cancer (MDA-MB-231); Fibroblasts (L929)	[4]
rGO/Fe_3O_4/Cu_2O NSs modified magnetic glassy carbon electrode	CV	230 pM	--	--	HeLa	[5]

References for table 1

(1) Hu, X. B.; Liu, Y. L.; Zhang, H. W.; Xiao, C.; Qin, Y.; Duo, H. H.; Xu, J. Q.; Guo, S.; Pang, D. W.; Huang, W. H. Electrochemical Monitoring of Hydrogen Sulfide Release from Single Cells. *ChemElectroChem* **2016**, *3*, 1998–2002.

(2) Asif, M.; Aziz, A.; Wang, Z.; Ashraf, G.; Wang, J.; Luo, H.; Chen, X.; Xiao, F.; Liu, H. Hierarchical CNTs@CuMn Layered Double Hydroxide Nanohybrid with Enhanced Electrochemical Performance in H 2 S Detection from Live Cells. *Anal. Chem.* **2019**, *91*, 3912–3920.

(3) Jeromiyas, N.; Mani, V.; Chang, P. C.; Huang, C. H.; Salama, K. N.; Huang, S. T. Anti-Poisoning Electrode for Real-Time in-Situ Monitoring of Hydrogen Sulfide Release. *Sensors Actuators, B Chem.* **2021**, *326*, 128844.

(4) Panda, A. K.; Keerthi, M.; Sakthivel, R.; Dhawan, U.; Liu, X.; Chung, R. J. Biocompatible Electrochemical Sensor Based on Platinum-Nickel Alloy Nanoparticles for In Situ Monitoring of Hydrogen Sulfide in Breast Cancer Cells. *Nanomater. 2022, Vol. 12, Page 258* **2022**, *12*, 258.

(5) Gu, W.; Zheng, W.; Liu, H.; Zhao, Y. Electroactive Cu2O Nanocubes Engineered Electrochemical Sensor for H2S Detection. *Anal. Chim. Acta* **2021**, *1150*, 338216.

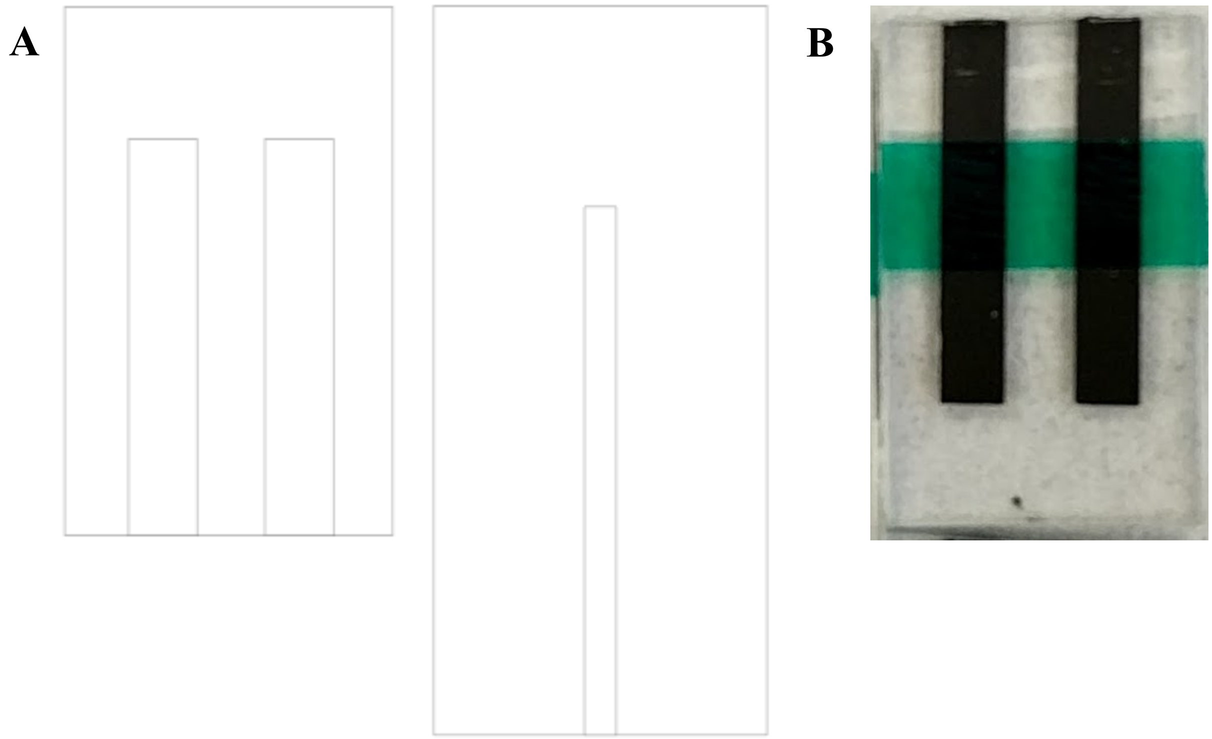

Figure S-1. Mask schemes for the two SPCE designs. (A) Vinyl mask designs. Left: dual electrode (3.2 mm x 19.0 mm) for analytical performance and selectivity measurements. Right: Single, thin electrode (1.6 mm x 25.4 mm) for cellular measurements. (B) Image of completed dual SPCE with Kapton tape (green) to demarcate working area (3.2 mm x 6.5 mm) from lead connections.

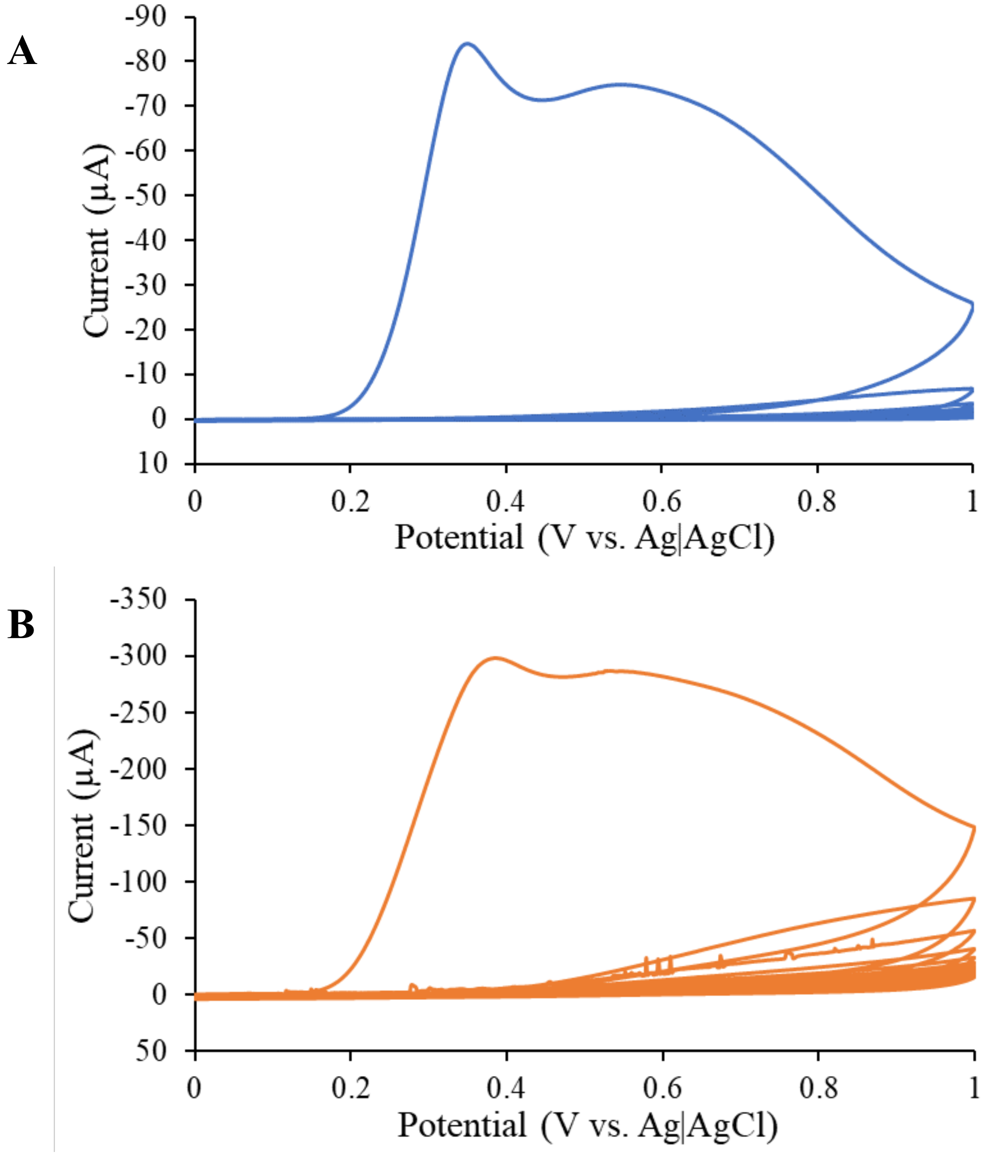

Figure S-2. Cyclic voltammograms of the electropolymerization of *o*-PD upon (A) glassy carbon electrodes and (B) SPCEs. Performed in 10 mM *o*-PD in 10 mM PBS, sweeping from 0.0 to +1.0 V at a scan rate of 10 mV s^{-1}. Initial sweep shows the characteristic peak at +0.4 V and prominent shoulder (+0.5 to +0.8 V). Subsequent sweeps are passivated by the deposited film.

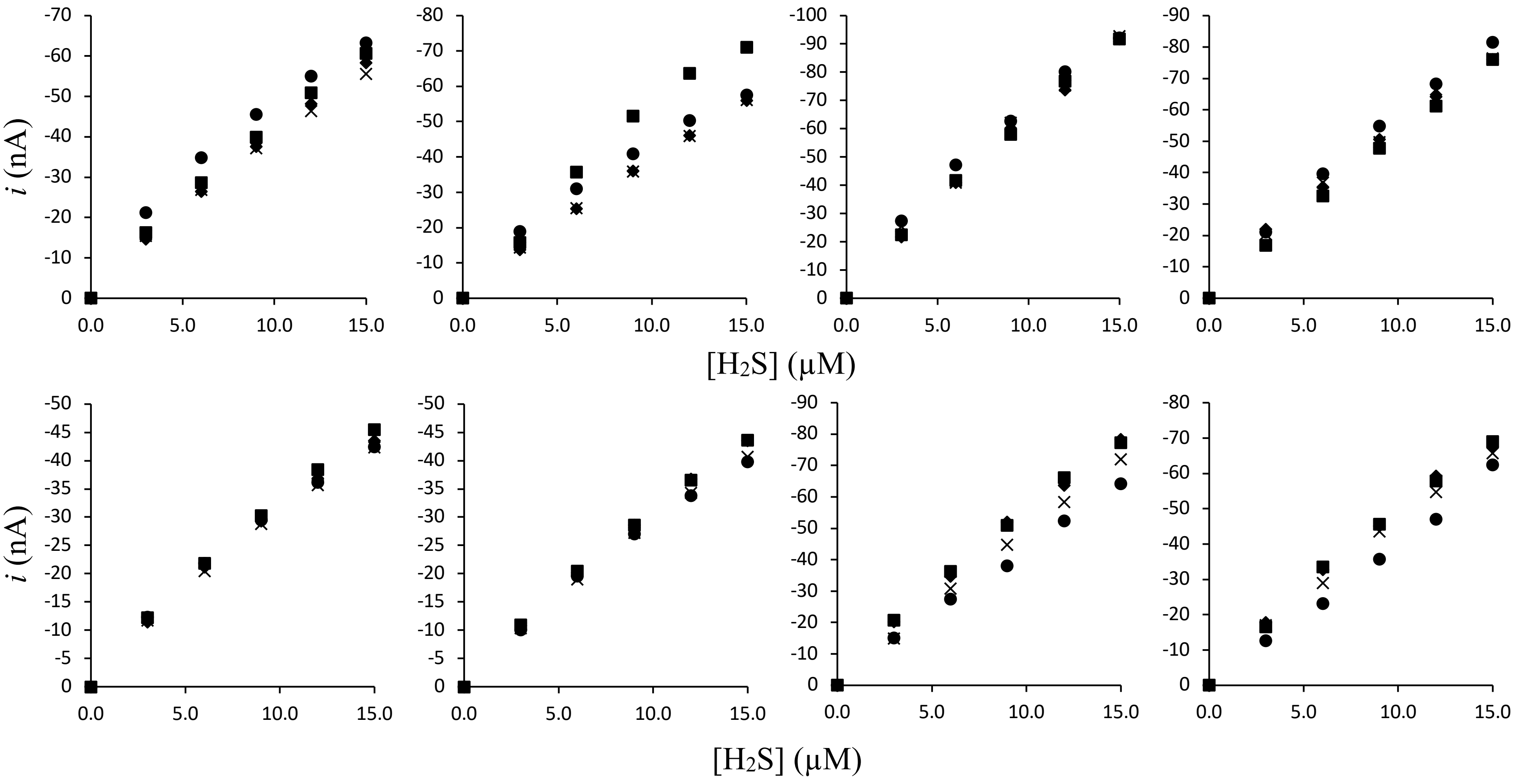

Figure S-3. Repeated standard calibrations performed on bare (Top row, N = 4) and poly-*o*-PD-coated (Bottom row, N = 4) SPCEs. Each graph is four consecutive standard calibrations performed using a single SPCE. Key for each graph: Calibration 1 (circle), Calibration 2 (X symbol), Calibration 3 (diamond), and Calibration 4 (square). All calibration curves had background current subtracted.
